# Erratum for Euro Surveill. 2018;23(20)

**DOI:** 10.2807/1560-7917.ES.2018.23.22.180531-1

**Published:** 2018-05-31

**Authors:** Eurosurveillance editorial team

**Affiliations:** 1 European Centre for Disease Prevention and Control (ECDC), Stockholm, Sweden

In the article ‘Establishment of the European meningococcal strain collection genome library (EMSC-GL) for the 2011 to 2012 epidemiological year’, published on 17 May 2018, the column headings in Table 3 were misaligned. The mistake was corrected on 31 May 2018.
